# The contribution of school breaks to the all-day physical activity of 9- and 10-year-old overweight and non-overweight children

**DOI:** 10.1007/s00038-012-0355-z

**Published:** 2012-03-14

**Authors:** Dorota Groffik, Erik Sigmund, Karel Frömel, František Chmelík, Petra Nováková Lokvencová

**Affiliations:** 1 The Jerzy Kukuczka Academy of Physical Education, Katowice, Poland; 2 Center for Kinanthropology Research, Faculty of Physical Culture, Palacky University in Olomouc, Olomouc, Czech Republic

**Keywords:** Obesity, School regime, Break, Steps, ActiTrainer, Heart rate

## Abstract

**Objectives:**

This study examines whether moderate-to-vigorous physical activity (MVPA), in at least 30-min school breaks (SB), helps to achieve the health-related amount of daily physical activity (PA) and whether these exercises influence after-school PA.

**Methods:**

The ActiTrainer-based PA was monitored over two school days in 239 children aged from 9 to 10 (57.3% female; 20.1% overweight, and 19.2% obese), in Katowice, in February 2010. PA was assessed based on steps, heart rate, and duration of PA.

**Results:**

MVPA, for 30 min during SB, represented an average of 1,258 steps for overweight girls and 1,620 steps for boys, and 1,336 steps for non-overweight girls and 1,758 steps for boys. Children with 30 min of MVPA during SB attained a higher daily amount of steps (*p* < 0.001) and duration of overall PA (*p* < 0.01), in comparison with less physically active children.

**Conclusion:**

The daily 30 min of MVPA during SB amounts to 12.5% of the overall number of steps for girls and 16.3% for boys, thus contributing to higher school PA and overall PA and leading to the achievement of the health-related minimum of PA.

## Introduction

The worldwide growth in childhood obesity (Apfelbacher et al. 2008; Stamatakis et al. 2010) in connection with a corresponding decline in physical activity (PA) and inappropriate dietary patterns (Edwards et al. 2010; Janssen et al. 2005) has led to the need for open discussion regarding possible effective methods of increasing children’s daily PA. Schools represent an opportunity for implementing PA (Edwards et al. 2010; Sharma 2006) and nutrition intervention programs (Evans et al. 2010; Li and Hooker 2010; Ransley et al. 2007; Sharma 2006). As children spend much of their childhood in school (Fox 2004), the school environment can instigate and maintain healthy lifestyle habits (Pate et al. 2006; Sharma 2006). School-related PA (Guinhouya et al. 2009; Tudor-Locke et al. 2009a), including active school commuting (Faulkner et al. 2009; McKee et al. 2007; Panter et al. 2010), physical education (PE) lessons (Pate et al. 2006; Tudor-Locke et al. 2009a), and morning, lunchtime, and other PA breaks (Ridgers et al. 2009, 2010; Verstraete et al. 2006) have been helpful in effectively increasing the daily PA of school children (Mota et al. 2005; Pate et al. 2006).

The demonstrable health benefits of PA for children (e.g. reduction of high blood pressure, obesity and depression; increases in fitness and bone-mineral density) are brought about by an accumulation of an average of at least 60 min of PA per day and up to several hours of at least moderate intensity PA (Janssen and LeBlanc 2010; Strong et al. 2005). However, some of these health benefits can be achieved through an average of 30 min of moderate-to-vigorous PA (MVPA) per day (Janssen and LeBlanc 2010). While health-enhancing PA in adults needs to last for 20–60 min without a break, it can be carried out by children in shorter, 10- to 15-min intervals adding up to 60 min or more of MVPA per day (Strong et al. 2005). These shorter PA episodes should be primarily carried out in PE lessons (Cale and Harris 2006; Strong et al. 2005) and in break periods (Mota et al. 2005; Ridgers et al. 2010; Verstraete et al. 2006). In relation to these findings, medical experts have stated that schools should ensure that all children and youth partake in a minimum of 30 min of MVPA during each school day (Pate et al. 2006) and the total health-related minimum of steps should amount to 11,000 steps per day for girls and 13,000 for boys (President’s Council on Physical Fitness and Sports 2001; Vincent and Pangrazi 2002).

Previous studies demonstrate the appropriateness of the school environment for daily PA for children and the reduction of their sedentary behaviour. Potentially novel approaches for reducing children’s sedentary time include activity breaks during class time, delivery of active lessons and homework, realisation of non-elimination games and changes to the classroom environment and furniture (Foster et al. 2010; Salmon 2010). Permanent play facilities (Nielsen et al. 2010) are a proven determinant of increased PA for 9- and 10-year-old children at school. These involve not only playgrounds with adventure playgrounds, swings, trees, playground markings and sandpits, but also corridors, free classrooms, movement ‘corners’ and courts (Nielsen et al. 2010).

The present study attempts to uncover the potential of MVPA during school breaks for attaining the health-related amount of overall daily PA in 9- and 10-year-old overweight and non-overweight school children. This study assessed the contribution of 30 min of MVPA during school breaks to the overall daily PA and to the acquirement of health-related amount of steps in 9- and 10-year-old overweight and non-overweight school children. The specific objectives were to:

- Describe and compare differences in overall PA in the specific parts of the school day (before school, PE and another lessons, breaks, after school) in overweight and non-overweight girls and boys divided based on the duration of MVPA in school breaks.
- Find out and describe the relationship between school breaks and the overall daily PA in the groups of children stratified by gender and obesity levels.
- Describe and compare the intensity of overall PA during school breaks in overweight and non-overweight girls and boys divided based on the duration of MVPA during the school breaks.

Overall daily PA will be assessed according to variables simultaneously measured by the ActiTrainer monitor; variables include number of steps, heart rate and duration of PA. We expect that the children who partake in at least 30 min of MVPA during the school breaks will attain a higher amount of overall daily PA. However, we do not know to what extent the expected contribution would be in presently overweight children.

## Methods

### Participants and settings

Elementary schools were selected intentionally based upon uniformly implemented daily school routines, similar size and equipment, number of students and localization in the city’s development. All third grade classes from the selected schools were included in the study. Participants included 149 girls and 111 boys aged 9 and 10 in six classes of two elementary schools in Katowice (Poland) who returned signed, informed parental-consent forms enabling them to participate in the study. The final, entire 2-day monitored physical activity (using an ActiTrainer activity monitor) was completed, and 137 girls and 102 boys with a mean age of 9.48 ± 0.40 years (20.1% overweight and 19.2% obese) were included in the analysis of the data. Illnesses and non-participation in the physical education lesson were a reason for the elimination of 15 and 6 children, respectively (representing 8% of girls and 8% of boys). All of the children assessed wore the ActiTrainer activity monitor continuously for 2 days (excluding sleeping, hygiene, and bathing times) for a minimum of 10 h per day.

Between the 4th and 5th and the 11th and 12th of February 2010, all of the participating children followed their usual daily school routine, including five school lessons, four breaks and one lunchtime break. School lessons usually started at 8 a.m., were each 45 min in length and finished between 1 and 2 p.m. Each of the children participated in one PE lesson with the same gymnastics content. The lunchtime break was also 45 min in length. One of the four school breaks lasted 30 min, while the others were 5 min long. During the said 30-min break, the children played movement games, participated in rhythmic and dance activities as well as used sports and game equipment (e.g., a bouncy ball, frisbee, gymball, hopscotch and skipping rope) in the playground under the supervision of teachers. However, additional spontaneous PA was not restricted. The children remained in their classrooms during the shorter breaks or could move around spontaneously in the school corridors under the supervision of teachers.

The study was approved by the Institutional Research Ethics Committee of Palacky University. Children, parents and teachers participated in the study voluntarily and received no compensation.

### Instruments and procedure

The ActiTrainer (ActiTrainer™, Florida, USA) is small and light (8.6 × 3.3 × 1.5 cm; 53 g). It is a multi-functional device composed of a heart-rate monitor, tri-axis solid-state accelerometer, an electronic pedometer, an inclinometer, and an ambient light sensor. The recording of data can be viewed on the built-in display. Upon turning on the display panel, the ActiTrainer can monitor and continually store recorded data over a period of 7 days. The validity and reliability of the ActiTrainer-based step counting in non-laboratory conditions was verified with 20 non-obese university students (Neuls 2008).

A right hip-fixed ActiTrainer activity monitor continuously measured the PA of children in 15-s intervals for the entire body-wearing time. When monitoring PA, the ActiTrainer was secured at the waist using a neoprene pouch and an elastic belt. When collecting heart-rate data, the Polar chest strap was worn across the sternum. The overall measured PA was simultaneously represented by number of steps, heart rate and duration of PA variables. Children were classed as being physically active, as opposed to sedentary, when >25 counts per 15 s were recorded. Intensity zones for overall PA were set based on the percentage of the maximal age-related heart rate, i.e., 220-age (Edwards 2010). Moderate-to-vigorous physical activity was defined as heart rate above 60% of the maximum age-related heart rate in accordance with published studies (Edwards 2010; Kirkpatrick and Birnbaum 1997).

Age was computed from the date of birth and the date of monitoring. Children’s height was measured using an Anthropometer A-319 (Trystom Corporation, Olomouc, Czech Republic) while their body weight was measured using calibrated Tanita WB 110 S MA (Quick Medical Corporation, Seattle, WA, USA). The first author measured the height and weight to the nearest 0.5 cm and 0.1 kg. BMI was calculated as body mass in kilograms divided by height in meters squared (kg/m^2^). Children were classified as overweight if their BMI was equal to or greater than the sex- and age-specific 85th percentile from the World Health Organization growth charts (World Health Organization 2007) available from the World Health Organization web site.

After completing morning hygiene routines on the first day, the monitored children’s parents fastened the Polar chest strap (Wearlink T31) around their child’s chest and fitted the elastic waist belt with the ActiTrainer on their right hip. After arriving at school, the first author of the study and the class’ teachers checked the functioning of the ActiTrainer and wrote down the time of arrival in the proxy report. Teachers further recorded the beginning times of the school lessons and breaks in the proxy report. In the evening, the parents recorded the time at which both elastic belts were removed.

### Statistical analyses

Statistical analyses including descriptive, correlations, and analyses of variance were performed using SPSS 19.0. A series of one-way (at least 30 min or less than 30 min of moderate-to-vigorous PA during school breaks) ANOVAs were conducted to examine possible differences in overall PA (number of steps, heart rate, duration of PA) in each part of the school day, separately for girls and boys. Before school time, PE lessons, teaching lessons, breaks, and after-school time were used as the dependent variables. A comparison of the proportion of overweight and obese children with and without 30 min of moderate-to-vigorous PA during school breaks was carried out using Mann–Whitney *U* non-parametric tests. Three-time and three-way (gender; overweight and non-overweight state; at least 30 min or less than 30 min of MVPA during school breaks) ANOVAs were used to examine the strength of the tested factors for overall daily PA. Spearman rank correlation *r*_S_ was used to determine the relationship between school breaks and the overall daily PA. Three-time and three-way (gender; overweight and non-overweight state; at least 30 min or less than 30 min of moderate-to-vigorous PA during school breaks) ANOVAs were also used to determine the variation in the intensity of overall PA during school break time in the three heart-rate zones. In order to identify the differences in overall PA between overweight and non-overweight girls and boys with or without 30 min of MVPA, a post hoc Scheffe test was used. The estimate of the strength of the relationship between the independent and dependent variables was represented as coefficients effect size “*d*” for ANOVAs (Cohen 1988) and the Mann–Whitney test (Cortina and Nouri 2000). The values of 0.2, 0.5, and 0.8 were interpreted as small, medium, and large effects, respectively (Thomas and Nelson 2001).

## Results

The mean (SD) values for the children’s anthropometric characteristics and physical activity levels during the segmented school day are presented in Table 1. Of the participants, 42 girls (30.6%) and 52 boys (51%) were classified as overweight or obese. Children of both gender, with at least 30 min of moderate-to-vigorous school-break PA, show a significantly higher number of steps and duration of PA at school (*p* < 0.001) and overall daily PA (*p* < 0.01) than children without 30 min of MVPA during school breaks (Table 1). A higher heart rate at school (*p* < 0.001) and overall daily PA (*p* < 0.01) in girls with at least 30 min of MVPA school breaks was also found when compared with girls with less than 30 min of MVPA during school breaks (Table 1).

**Table 1:** Anthropometric characteristics and physical activity levels [mean (standard deviation)] of 9- and 10-year-old overweight and non-overweight children divided based on the duration of the moderate-to-vigorous physical activity in school breaks (Katowice, Poland, 2010)

	Girls (*n* = 137)			Boys (*n* = 102)		
	≥30 min of MVPA breaks (*n* = 65)	Effect size (*d*)	<30 min of MVPA breaks (*n* = 72)	≥30 min of MVPA breaks (*n* = 53)	Effect size (*d*)	<30 min of MVPA recess (*n* = 49)
Anthropometric data						
Age (years)	9.36 (0.38)		9.62 (0.34)	9.35 (0.38)		9.56 (0.44)
Body height (cm)	134.62 (6.03)	0.29	136.64 (7.62)	136.11 (5.84)	0.18	135.02 (6.62)
Body weight (kg)	32.92 (8.01)	0.30	35.39 (8.40)	35.38 (6.83)	0.22	33.74 (8.09)
BMI (kg/m^2^)	17.99 (3.03)	0.10	18.69 (4.21)	19.04 (3.03)	0.29	18.32 (3.23)
Overweight^a^	*n* = 14 (21.5%)	0.16	*n* = 11 (15.3%)	*n* = 12 (22.6%)	0.04	*n* = 11 (22.5%)
Obesity^b^	*n* = 6 (9.2%)	0.18	*n* = 11 (15.3%)	*n* = 17 (32.1%)	0.21	*n* = 12 (24.5%)
Steps (number)						
Before school	1,219 (880)	0.48	844 (677)**	1,315 (860)	0.14	1,198 (857)
At school	3,049 (732)	1.81	1,500 (954)***	3,474 (1,152)	1.11	2,110 (1,298)***
PE lesson	1,134 (674)	0.20	1,244 (413)	1,218 (751)	0.49	1,544 (563)**
Another lessons	831 (532)	0.74	492 (379)***	894 (696)	0.23	742 (636)
Breaks	1,313 (406)	2.44	456 (294)***	1,683 (628)	2.06	643 (321)***
After school	7,089 (3,012)	0.09	6,779 (3,488)	6,644 (3,899)	0.45	5,141 (2,654)*
All day	11,357 (2,888)	0.67	9,124 (3,689)***	11,433 (4,057)	0.84	8,449 (2,890)***
Heart rate (beats/min)						
Before school	111.96 (17.13)	0.12	110.06 (14.22)	110.58 (18.92)	0.42	103.93 (11.75)*
At school	112.80 (9.74)	0.69	106.15 (9.63)***	108.59 (9.46)	0.16	107.06 (9.73)
PE lesson	128.36 (20.42)	0.46	136.20 (13.24)	122.34 (21.81)	0.62	134.12 (15.52)*
Another lessons	107.15 (10.13)	0.65	101.17 (8.13)***	102.58 (10.36)	0.17	100.97 (7.89)
Breaks	125.01 (11.13)	1.00	114.26 (10.32)***	122.77 (11.63)	0.16	120.73 (13.23)
After school	108.22 (10.45)	0.44	103.73 (9.83)*	104.04 (13.60)	0.24	101.17 (10.15)
All day	111.00 (9.90)	0.48	106.64 (8.14)**	107.74 (9.44)	0.43	104.05 (7.59)*
Duration of PA (min)						
Before school	42.17 (32.40)	0.54	27.21 (22.16)**	39.68 (23.86)	0.09	42.27 (31.84)
At school	135.80 (21.33)	2.18	81.33 (27.92)***	142.53 (27.81)	1.77	96.95 (23.27)***
PE lesson	30.49 (10.45)	0.44	34.38 (7.29)	30.12 (11.36)	0.79	36.77 (2.69)**
Another lessons	75.22 (17.72)	1.30	49.20 (21.88)***	83.15 (27.10)	0.89	60.31 (23.95)***
Breaks	38.52 (5.16)	3.77	16.85 (6.23)***	40.06 (5.58)	3.35	19.39 (6.75)***
After school	254.48 (66.78)	0.21	270.10 (77.91)	233.44 (82.27)	0.18	218.50 (87.90)
All day	431.07 (65.08)	0.68	378.64 (86.99)***	414.80 (91.48)	0.65	357.71 (83.68)**

*n* number of participants, *MVPA* moderate-to-vigorous physical activity, *BMI* body mass index, *PE* physical education, *PA* overall physical activity, *d* coefficients effect size for one-way analysis of variance (Cohen 1988) and the Mann–Whitney test (Cortina and Nouri 2000). The values of 0.2, 0.5, and 0.8 were interpreted as small, medium, and large effects, respectively (Thomas and Nelson 2001)

Statistical significance (one-way analysis of variance) of the differences between groups of children ≥30 min and <30 min of MVPA during school breaks is expressed as: * *p* < 0.05, ** *p* < 0.01, *** *p* < 0.001

^a^Overweight or ^b ^Obesity represents BMI from 85th to 97th or greater than 97th of WHO growth charts (World Health Organization 2007)

Three-way ANOVAs determined the significant effect of ≥30 min of MVPA during school breaks on the overall daily PA (number of steps *F* = 27.66, *p* < 0.001; heart rate *F* = 13.48, *p* < 0.001; duration of PA *F* = 19.40, *p* < 0.001). The effect of ≥30 min of MVPA during school breaks on overall daily PA is more significant than the two other tested factors, gender and overweight. In terms of gender, a significant effect was only found on all-day PA with heart rate (*F* = 6.37, *p* = 0.012); with the overweight variable, a significant effect was only found with the number of steps (*F* = 4.45, *p* = 0.036). Overweight and non-overweight girls and boys with at least 30 min of MVPA during school breaks attained more than 10,000 steps per day. Overweight and obese children with at least 30 min of MVPA during school breaks demonstrated about 2,362 more steps per day than overweight and obese children with less than 30 min of MVPA during school breaks. Taken as a whole, girls and boys with at least 30 min of MVPA during school breaks were more active by more than 2,200 steps per day when compared with girls and boys with less than 30 min of MVPA during school breaks.

PA during PE lessons has a significant effect (*p* < 0.001) on overall daily PA represented by the number of steps, the heart rate, and the duration of PA. The effect of the realisation of PA during the PE lesson on overall daily PA was more significant than the other two factors, gender and overweight.

The length of school-break PA is positively associated in a significant fashion with the overall daily PA of girls (number of steps *r*_S_ = 0.36, *p* < 0.001; heart rate *r*_S_ = 0.55, *p* < 0.001; duration of PA *r*_S_ = 0.30, *p* < 0.001) and boys (number of steps *r*_S_ = 0.50, *p* < 0.001; heart rate *r*_S_ = 0.49, *p* < 0.001; duration of PA *r*_S_ = 0.29, *p* = 0.004). The positive relationship between PA during school breaks and overall daily PA is slightly higher with non-overweight children (number of steps *r*_S_ = 0.41, *p* < 0.001; heart rate *r*_S_ = 0.46, *p* < 0.001; duration of PA *r*_S_ = 0.33, *p* < 0.001) than in their overweight peers (number of steps *r*_S_ = 0.38, *p* < 0.001; heart rate *r*_S_ = 0.55, *p* < 0.001; duration of PA *r*_S_ = 0.21, *p* = 0.05).

**Fig. 1:**
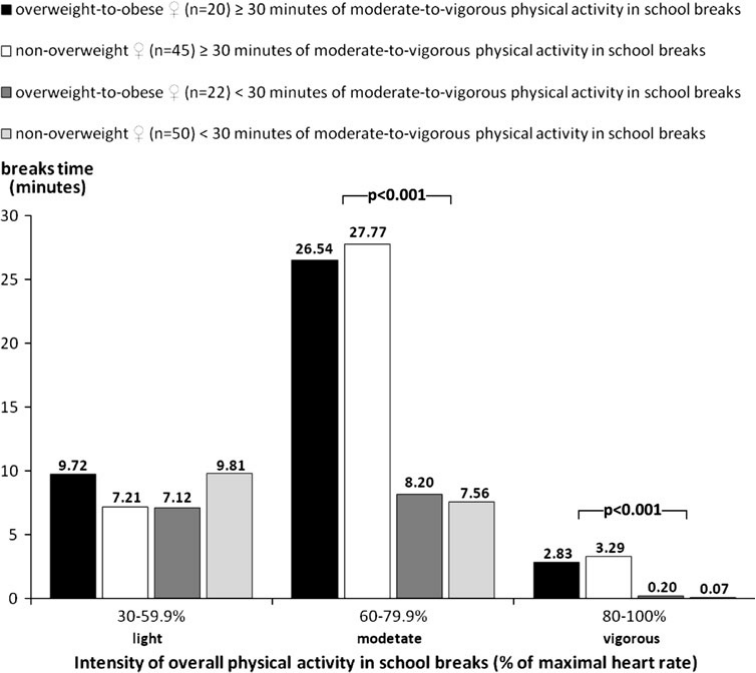
Comparison of the intensity of overall physical activity in school breaks time for 9- and 10-year-old overweight and non-overweight girls divided based on the duration of moderate-to-vigorous physical activity during school breaks (Katowice, Poland, 2010). *p* level of statistical significance (post hoc Scheffe test), maximum heart-rate values were calculated by a simplified age-related Karvonen equation, i.e., 220-age (Edwards 2010)

**Fig. 2:**
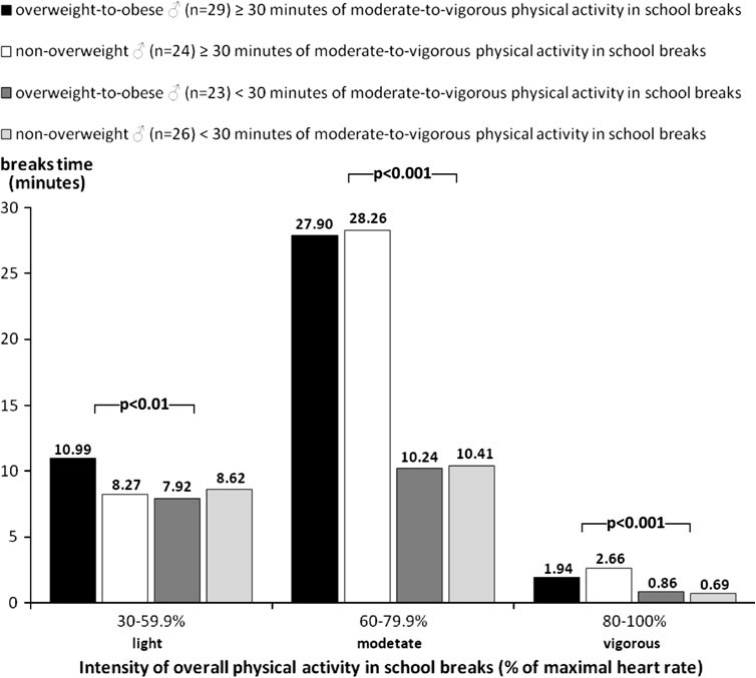
Comparison of the intensity of overall physical activity during school breaks for 9- and 10-year-old overweight and non-overweight boys divided based on the duration of moderate-to-vigorous physical activity during school breaks (Katowice, Poland, 2010). *p* level of statistical significance (post hoc Scheffe test), maximum heart-rate values were calculated by a simplified age-related Karvonen equation, i.e., 220-age (Edwards 2010)

The distribution of the intensity of overall PA in school breaks for overweight and non-overweight girls and boys is presented in Figs. 1 and 2. Both, overweight and non-overweight children with at least 30 min of MVPA during school breaks demonstrated a significantly longer period of PA in moderate (*F* = 93.85, *p* < 0.001, *d* = 1.33) and vigorous (*F* = 19.88, *p* < 0.001, *d* = 0.59) heart rate zones than children with less than 30 min of MVPA during school breaks (Figs. 1, 2).

## Discussion

This study indicated that school-related physical activity (PE lessons and break-time activities) represent a substantial part of the daily PA of school children (Loucaides and Jago 2008; Pate et al. 2006; Ridgers et al. 2009, 2010). Because of similarities in the results of the PA variables (the number of steps, heart rate, and duration of PA) of the participants, this discussion will concentrate on the particularly frequent variable, number of steps. In support of Tudor-Locke et al. (2009a) and Loucaides and Jago (2008), we have confirmed that overweight and non-overweight girls take an average of 24.1 and 22.2% of the daily steps within school, 11.1 and 10.8% during PE lessons, and 9.4 and 8.5% during breaks. Likewise, overweight and non-overweight boys reach 29.8 and 30.4% of the daily steps within school, 10.5 and 15.1% during PE lessons, and 12.2 and 12.5% at breaks. This study highlighted the considerable contribution of at least 30 min of moderate-to-vigorous school-break PA to daily steps for overweight and non-overweight children (15.1 and 13.6%). PA realised in PE lessons represents a similar contribution (>13%) to the total number of daily steps in overweight and non-overweight children.

During breaks between school lessons, children were able to move about spontaneously in the school corridors or carry out both structured and unstructured PA in the playground under the supervision of teachers. Structured PA consisted of the playing of movement games, rhythmic and dance activity as well as using sports and game equipment. The children, irrespective of sex or body-weight status, made use of the opportunity to be active. Their involvement in the PA was most clearly marked in activities of a moderate intensity. Children who were not interested in participating in PA during school breaks were offered PA, but were not forced to participate. Previous studies (Ridgers et al. 2007; Sallis et al. 2001; Stratton and Mullan 2005; Verstraete et al. 2006) corroborate the present study, illustrating that PA-friendly school environments (Stratton and Mullan 2005; Ridgers et al. 2007) with high levels of equipment (Verstraete et al. 2006) and supervision (Sallis et al. 2001) stimulated children to be more active, irrespective of sex (Sallis et al. 2001; Verstraete et al. 2006) or body-weight status.

Participation in at least 30 min of MVPA during school breaks significantly contributes to an increase in overall school- and all-day PA in overweight and non-overweight girls and boys and assists in approaching the health-related minimum of daily amount of steps (11,000 and 13,000 steps per day for girls and boys) for this age category of children (President’s Council on Physical Fitness and Sports 2001; Vincent and Pangrazi 2002).

Female participants who partook in longer sessions of MVPA during school breaks also demonstrated a higher level of before-school PA. Girls with at least 30 min of MVPA during school breaks had a before-school number of steps equating to 10.7% of their all-day steps, while girls with less than 30 min of MVPA during school breaks had before-school steps of only 9.3%. Before-school PA is undoubtedly linked with active school commuting, which is considered another effective way of increasing daily PA for school children (Faulkner et al. 2009; McKee et al. 2007; Panter et al. 2010).

## Limitations

The study’s strengths include the triangulation approach to the assessment of PA levels. Three different variables (number of steps, duration of PA, and heart rate) from one ActiTrainer monitoring device provide more reliable results than results based on one variable alone.

However, this study has three main limitations. First, it conducted a simplified calculation of the maximum age-related heart rate without knowledge of individuals’ resting heart rates. Additionally, a possible ‘ActiTrainer wear’ reactivity effect could influence the results, although two-day instrument monitoring of free-living PA is considered non-reactive (Tudor-Locke et al. 2009b). For a more accurate measurement of the level of PA in the field using an ActiTrainer monitor, a longer period of monitoring (including at least one weekend day) in different seasons of the year would be needed. A more comprehensive understanding of the overall daily PA of school-age children also requires valid information as to the possible influence of lifestyle behaviour, parents and peer support, and the environmental characteristics of their residence.

## Conclusion

The prevalence of obesity and decreased PA among school-age children underpinned the implementation of effective strategies to increase daily PA and counteract child obesity. Schools represent an ideal environment for implementing physical activity programs and strategies. School-related PA, including PE lessons and morning, lunchtime, and other breaks involving PA, has been helpful in terms of increasing the daily PA of school children. Pilot findings imply that 30 min of daily moderate-to-vigorous PA during school breaks significantly contributes to higher school and overall daily PA of overweight and non-overweight 9- and 10-year-old children and assists in the attainment of the health-related minimum of PA. Monitoring children’s all-day PA with the use of the ActiTrainer multifunctional device made possible a comprehensive analysis and assessment of children’s behaviour.

To conclude, promotion of an active lifestyle in children and youth is one of the health priorities of developed countries. Involvement of schools in the implementation of such priorities seems to be effective. By facilitating PA during school breaks, it is possible to encourage children to have an active lifestyle without regard to their gender, race or obesity level.
